# Surveillance of the Remaining Nodules after Resection of the Dominant Lung Adenocarcinoma is an Appropriate Follow-Up Strategy

**DOI:** 10.3389/fsurg.2014.00052

**Published:** 2015-01-12

**Authors:** Massimo Castiglioni, Brian E. Louie, Candice L. Wilshire, Alexander S. Farivar, Ralph W. Aye, Jed Gorden, Matthew P. Horton, Eric Vallières

**Affiliations:** ^1^Center for Thoracic Surgery, University of Insubria, Varese, Italy; ^2^Division of Thoracic Surgery, Swedish Cancer Institute, Seattle, WA, USA; ^3^Cellnetix Pathology, Seattle, WA, USA

**Keywords:** lung adenocarcinoma, lepidic growth pattern, multinodular disease, surveillance, additional nodules

## Abstract

**Introduction:** Adenocarcinomas, commonly present as a dominant lesion (DL) with additional nodules in the ipsilateral or contralateral lung. We sought to determine the fate and management of the secondary nodules and to assess the risk of these nodules using the Lung CT Screening Reporting and Data System (Lung-RADS) criteria and the National Comprehensive Cancer Network (NCCN) Guidelines to determine if surveillance is an appropriate strategy.

**Methods:** We retrospectively evaluated patients with lepidic growth pattern adenocarcinoma and secondary nodules from 2000 to 2013. Risk assessment of the additional lesions was completed with a simplified model of Lung-RADS and NCCN-Guidelines.

**Results:** Eighty-seven patients underwent resection of 87 DLs (Group 1) concurrently with 60 additional pulmonary nodules (Group 2), while 157 non-DLs were radiologically surveyed over a median follow-up time of 3.2 years (Group 3). Malignancy was found in 29/60 (48%) nodules in Group 2. Whereas, only 9/157 (6%) of the lesions in Group 3 enlarged, 4 of which (2.5% of total) were found to be malignant, and then treated, while the remaining nodules continued surveillance. After applying the Lung-RADS and NCCN simplified models, nodules in Group 2 were at higher risk for lung cancer than those in Group 3.

**Conclusion:** In patients with lepidic growth pattern adenocarcinoma associated with multiple secondary nodules, surveillance of the remaining nodules, after resection of the DL, is a reasonable strategy since these nodules exhibited a slow rate of growth and minimal malignancy. In contrast, nodules resected from the ipsilateral lung at the time of the DL, harbor malignancy in 48%. Risk assessment models may provide a useful and standardized tool for clinical assessment of pulmonary nodules.

## Introduction

Advances in computed tomography (CT) as well as the increasing number of lung cancer screening programs has led to an increase in the detection of pulmonary nodules. The prevalence of multiple small lung nodules in the initial Early Lung Cancer Action Program (ELCAP) study (1) was 23% and increased to 50% when scans were performed with a thinner slice thickness (2). As a result, many lung cancers present as a dominant lesion (DL) with 40–57% having multiple secondary nodules particularly in the contralateral lung (3–6). Moreover, invasive adenocarcinomas, particularly with a lepidic component, have been associated with multiple nodules. After resection of the DL, the management of the additional lung nodules is quite varied and can include surgical resection, systemic therapy, radiation, and surveillance (7). This creates a challenge in clinical practice decision making since no standardized treatment algorithm exists.

Several algorithms such as International Early Lung Cancer Action Program (I-ELCAP) and National Comprehensive Cancer Network (NCCN) have been used to assess the importance of nodules identified during routine follow-up after identification or lung cancer screening (8, 9). These systems take into consideration the size of the different nodules, their morphology, and the changes in size of the nodules but none have been utilized to assess the risk of secondary nodules prior to and after cancer resection. The aims of this study were (1) to determine the fate of the nodules identified prior to and after resection of the dominant lung adenocarcinoma; (2) to determine the management of the secondary nodules; and (3) to retrospectively assess the risk of these nodules using the Lung CT Screening Reporting and Data System (Lung-RADS) criteria (8) and the NCCN Guidelines (9) after lung cancer surgery.

## Materials and Methods

We performed a retrospective chart and pathologic review from 2000 to 2013 of patients with lung adenocarcinoma with lepidic features presenting with additional lung nodules. Patients were primarily identified from the Division of Thoracic Surgery database and consequently confirmed as having “BAC” or adenocarcinoma with a “BAC” component in the Cellnetix Pathology database. The Swedish Medical Center Institutional Review Board approved the study protocol. Individual consent was waived due to the retrospective nature of the study.

We initially identified 103 patients who presented with multiple lung nodules. All patients underwent resection of the DL and were evaluated as having “BAC” or adenocarcinoma with a “BAC” component on pathology. The DL was defined as the one for which the workup and surgery were performed. More specifically, the DL was a new lesion clinically suspicious for malignancy, or a lesion which on follow-up that had either enlarged in total size, developed or increased its solid component, and/or was PET positive (SUV >2.5) (10). Patients were defined as having multinodular disease when the radiologist reported at least one lesion in addition to the DL on the pre-operative imaging studies. We excluded 16 patients after careful review of their charts: 4 with clinical stage IV at the time of presentation, 4 having the DL with maximum diameter >5 cm, 3 with clinical N1 or N2 disease, and 5 with mucinous adenocarcinoma cell type. Thus, 87 patients were included in the review and staged according to the 7th edition of the TNM staging system (11).

Lung nodules in this study were defined as focal non-linear opacity on chest imaging. They were characterized by size, location, and morphology (solid, semi-solid, or non-solid). All non-calcified nodules identified were included in the study but a positive finding on the CT scan was a nodule measuring 6 mm or more in the mean diameter (12). Nodules were placed into one of three groups: Group 1 (G1) comprises the primary or DL; Group 2 (G2) includes nodules resected concomitantly with the DL or within 3 months of the DL resection; and Group 3 (G3) includes any nodule that was identified on the pre operative scan immediately prior to resection of the DL and was followed over time. When the additional nodules were risk assessed, nodules in G2 located in the same lobe as the DL were excluded since the strategy of management for these nodules is less controversial.

We performed a multi-disciplinary review of the resected secondary nodules to assess the histology using Martini and Melamed criteria (13). Second primary or synchronous cancers were decided if during evaluation of the specimen atypical adenomatous hyperplasia (AAH) was identified and/or the secondary lesion was associated with a lepidic pattern at the edge of the lesion. Also, synchronous cancer was denoted if cell type and morphology was different than the primary. In the case of contralateral lesions, the mediastinal nodes were required to be uninvolved with cancer in addition to the previous criteria. A metastatic focus was denoted if morphology and cell type was similar to the primary.

After resection of the DL, patients were followed-up on an outpatient basis for surveillance and evaluation of recurrent disease at 4 monthly intervals for the first 2 years, 6 monthly intervals for the next 3 years, and then annually after 5 years. Patients would undergo at least 1 annual CT scan with or without contrast alternating with chest radiography, unless new findings dictated further investigation. Co-existing nodules and any new nodules were managed largely according to the I-ELCAP Algorithm and Guidelines (12).

The overall growth of nodules was radiologically assessed by the volume doubling time (VDT) measurement, which was calculated using the following equation based on the modified Schwartz formula (14).

$$\mathrm{VDT}=\frac{\left[\mathrm{Log}\;2\times t\right]}{\left[\mathrm{Log}\;\left(V_{2}∕V_{1}\right)\right]}$$

*t* is the interval, measured by days, between the two CT scans used for the assessment of nodule growth; while *V*_1_ and *V*_2_ are the initial and final nodule volume, respectively. Nodule volume (*V*) = π/6(ab^2^), where *a* is the longest horizontal axis and *b* is the maximum perpendicular diameter (15).

Each nodule was risk assessed by the following systems with the baseline obtained on the CT scan immediately prior to surgery, at each CT scan after surgery with the scan that identified a change being recorded, and if a change in the nodule was identified if any further testing such as PET, biopsy, or short interval CT was performed. For nodules that enlarged we reported assessment at baseline (Time 1), which means at the time of primary surgery when the DL was resected, at the specific follow-up time when lesions were found to be enlarged (Time 2) and then after additional diagnostic studies were performed due to suspicion of lung cancer (Time 3). The term “diameter” in both models refers to the mean of the longest diameter of the nodule and its perpendicular diameter.

First, the “risk” of each nodule was evaluated by assessing the additional nodules with a simplified model of either Lung-RADS assessment categories (Table 1) (8). According to the Lung-RADS system, we first applied the size thresholds to nodules that were either identified at the pre-operative CT scan as at baseline CT screening or found to be enlarged at the follow-up CT scan. We then classified lesions into categories 2, 3, 4A, and 4B. When previous CT films or reports were available for the comparison, lesions were classified using further descriptors by the same model based on the presence of any nodule growth either overall or of the solid component. Nodule growth was defined as an increase in size of more than 1.5 mm of the mean diameter.

**Table 1:** **Simplified models of lung-RADS assessment categories and NCCN Guidelines for lung cancer screening**.

Model	Size thresholds	Category	Descriptor	Probability of malignancy	Recommendation
**Lung-rads**	–	0	Incomplete	–	–
	–	1	Negative	<1%	Annual follow-up
	*Solid nodules:* <6 mm	2	Benign appearance	<1%	Annual follow-up
	*Part-solid:* <6 mm	
	*Non-solid:* <20 mm	
	*Solid nodules:* ≥6 to <8 mm or new 4 to <6 mm	3	Probably benign	1–2%	6-month follow-up
	*Part-solid:* ≥6 mm total diameter (TD) with solid component (SC) <6 mm or new <6 mm TD	
	*Non-solid:* ≥20 mm or new	
	*Solid nodules:* ≥8 to <15 mm or new 6 to <8 mm or growing <8 mm	4A	Suspicious	5–15%	3-month follow-up
	*Part-solid:* ≥6 mm TD with SC ≥6 to <8 mm or with a new or growing <4 mm SC	
	*Solid nodules:* ≥15 mm or new or growing and ≥8 mm	4B	Suspicious	>15%	Consider tissue sampling
	*Part-solid:* SC ≥8 mm or a new or growing ≥4 mm SC	
**NCCN Guidelines**	Solid or part-solid nodules: <6 mm	A	–	–	Annual follow-up
	Non-solid: ≤5 mm	
	Solid or part-solid nodules: 6–8 mm	B	–	–	3 to 6-month follow-up
	Non-solid: >5–10 mm	
	Solid or part-solid nodules: >8 mm	C	–	–	Consider tissue sampling
	Non-solid: >10 mm	

*Italics font is used to differentiate morphology of the nodule*.

Second, the “risk” of each nodule was also assessed by the NCCN Guidelines for lung cancer screening (Table 2) (9). Size thresholds for solid, part-solid, and non-solid nodules were applied to each lesion. In order to simplify the analysis as well as the interpretation of results, lesions were grouped into A, B, and C NCCN categories. Category A includes nodules for which an annual repeat CT screening was recommended while Category C includes those for which a more intensive surveillance and aggressive work-up was recommended. Finally, Category B is associated to an intermediate degree of recommendation.

**Table 2:** **Demographic features of 87 patients**.

Characteristic	No. (%)
Age year, mean (SD)	69 (10)
Gender
Male	14 (16)
Female	73 (84)
Ethnicity
Caucasian	73 (84)
Asian	7 (8)
Other	7 (8)
Smoking history
Positive	68 (78)
PY, median (IQR)	26 (2–40)
Negative	19 (22)
Positive history of cancer (any site)	40 (46)
Lung adenocarcinoma	4 (5)
Symptoms at diagnosis
Yes	21 (24)
No	66 (76)
Pulmonary function, median (IQR)
FEV1 as % of predicted	90 (79–104)
DLCO as % of predicted	77 (64–87)
ECOG performance status 0–1	86 (99)
ASA score, median (IQR)	3 (2–3)
No. of lesions per patients, median (IQR)	2 (1–4)
Laterality per patient
Unilateral disease	22 (25)
Bilateral disease	65 (75)
Pathologic TNM stage (7th Ed.)
IA	58 (67)
IB	20 (23)
IIA	5 (6)
IIIA	4 (5)

Chi-square test was used to compare categorical variables. Student’s *t*-test was used for single comparisons of continuous variables and ANOVA test for multiple comparisons. A *p* value <0.05 was considered significant. Statistical analyses were performed using the SPSS 19.0 statistical software package (SPSS Inc., Chicago, IL, USA).

## Results

A total of 87 patients with 304 nodules were included. Demographic characteristics of the patient cohort are demonstrated in Table 2. Patients were predominantly women (84%), Caucasian (84%), with a positive smoking history (78%) and had a mean age at diagnosis of 69 years. The majority of patients had bilateral disease at the time of presentation with a median number of additional nodules per patient of 2 (IQR 1–4).

Group 1 includes the 87 DLs that were resected, while Group 2 includes 60 additional nodules that were resected in addition to the DL: 57 were concomitantly removed and 3 were subsequently removed from the contralateral lung at a subsequent additional operation within 3 months of the initial DL surgery. Finally, Group 3 includes 157 additional nodules that were radiologically observed. The radiological features of the nodules within three groups are showed in Table 3. Nodule size, the prevalence of mixed morphology and PET avidity were higher in Group 1 compared to Group 3, while the proportion of nodules with non-solid or solid morphology was greater in the Group 3. Additionally, the VDT of nodules progressively increased from Group 1 to Group 3. The majority of additional nodules that were resected in Group 2 were located in the same lobe (65%) as the DL. The majority of nodules in Group 3 were contralateral (65%).

**Table 3:** **Radiological features of the 304 nodules stratified by groups**.

Characteristic	No. (%)			*p* Value
	Group 1	Group 2	Group 3	
No. of nodules	87 (29)	60 (20)	157 (52)	
Size of the nodules
Median diameter (IQR) (cm)^a^	2 (1.5–2.9)	0.9 (0.5–1.3)	0.5 (0.3–0.6)	(1, 2) < 0.0001
				(1, 3) < 0.0001
				(2, 3) < 0.0001
Mean diameter <0.6 cm^b^	1 (1)	19 (32)	113 (72)	<0.0001
Mean diameter ≥0.6 cm^b^	86 (99)	41 (68)	44 (28)	
Morphology
Non solid	12 (14)	17 (28)	57 (36)	<0.0001
Part solid	70 (80)	14 (23)	9 (6)	
Solid	5 (6)	29 (48)	91 (58)	
PET avidity (SUV >2.5)^c^	33 (43)	4 (7)	2 (1)	<0.0001
Location, regarding the DL
Same lobe	–	39 (65)	7 (4)	<0.0001
Different ipsilateral lobe	–	18 (30)	48 (31)	
Contralateral	–	3 (5)	102 (65)	
VDT in days, median (IQR)	546 (346–745)	813 (460–922)	1110 (655–1339)	(1, 2) 0.790
				(1, 3) 0.003
				(2, 3) 0.096

*^a^Median size of the nodules refers to the maximum diameter*.

*^b^Mean diameter is an average of length and width, according to the International Early Lung Cancer Action Program (I-ELCAP) protocol (16)*.

*^c^PET-scan was available for 76 nodules in Group 1, 56 in Group 2, and 136 in Group 3*.

Dominant lesions were resected by lobectomy, segmentectomy, and wedge resection in 55, 17, and 28% of cases, respectively. Also, 60 additional nodules were resected, including 57 ipsilateral nodules concomitantly removed with the DL. Specifically, 36/57 (63%) lesions were in the same lung tissue of the resected DL, while 21/57 (37%) lesions required additional procedure, mainly a wedge resection and a segmentectomy in two cases. Three contralateral nodules were resected by a wedge resection at a subsequent operation 3 and 8 weeks after resection of the DL.

All resected DLs in Group 1 were malignant, mainly consisting of well or moderately well differentiated IA (Table 4). In Group 2, 39 nodules were in the same lobe as the DL and resected, while 18 were in the remaining ipsilateral lobes and 3 in the contralateral lung and they were also resected. Of the 39 nodules in the same lobe, 19 (49%) were found to be malignant whereas 10/21 (48%) of the nodules in the other ipsilateral or contralateral lobes were malignant. In Group 3, nodules were followed for a median 3.2 years (IQR 1.8–6.0). Of these, 9/157 (6%) nodules enlarged (Table 5) and four of which (2.5% of total) were found to be malignant. Three were resected via wedge resection and were found to be malignant, all consistent with IA. One was treated with stereotactic body radiation therapy. The remaining 153 lesions continue to be monitored.

**Table 4:** **Pathologic features of resected nodules within three groups**.

Characteristic	No. (%)				*p* Value
	Group 1 (*n* = 87)	Group 2		Group 3 (*n* = 157)	
		(*n* = 21)^a^	(*n* = 39)^b^	
No. of resected nodules	87 (100)	21 (100)	39 (100)	3 (2)	<0.0001
Benign histology	0 (0)	11 (52)	20 (51)	–	<0.0001
Malignant histology	87 (100)	10 (48)	19 (49)	3 (100)	
AIS	16 (18)	3 (30)	7 (37)	–	–
MIA	19 (22)	–	–	–	
IA	52 (60)	7 (70)	10 (53)	3 (100)	
Metastases	–	–	2 (11)	–	
Histologic grade
1	37 (43)	4 (40)	7 (37)	1 (33)	–
2	41 (47)	4 (40)	5 (26)	2 (66)	
3	9 (10)	–	3 (16)	–	
4	–	–	–	–	
Unknown	–	2 (20)	4 (21)	–	

*AIS, adenocarcinoma *in situ*; MIA, minimally invasive adenocarcinoma; IA, invasive adenocarcinoma*.

*^a^Only nodules located in a different ipsilateral lobe as the DL or contralaterally*.

*^b^Only nodules located in the same lobe of the DL*.

**Table 5:** **Characteristics of the additional 157 nodules that were radiologically observed**.

Characteristics	No. (%)		*p* Value
	Enlarged lesions	Unchanged lesions	
No. of lesions	9 (6)	148 (94)	–
Location
Same lobe	–	7 (5)	0.965
Different ipsilateral lobe	3 (33)	45 (30)	
Contralateral lung	6 (67)	96 (65)	
Morphology
Non-solid	–	57 (39)	<0.0001
Part-solid	7 (78)	2 (1)	
Solid	2 (22)	89 (60)	
Malignant histology	4 (44)	–	–
IA	3 (75)	–	–
NSCLC^a^	1 (25)	–	

*^a^Nodule biopsy was performed by CT-guided FNA only*.

**Figure 1:**
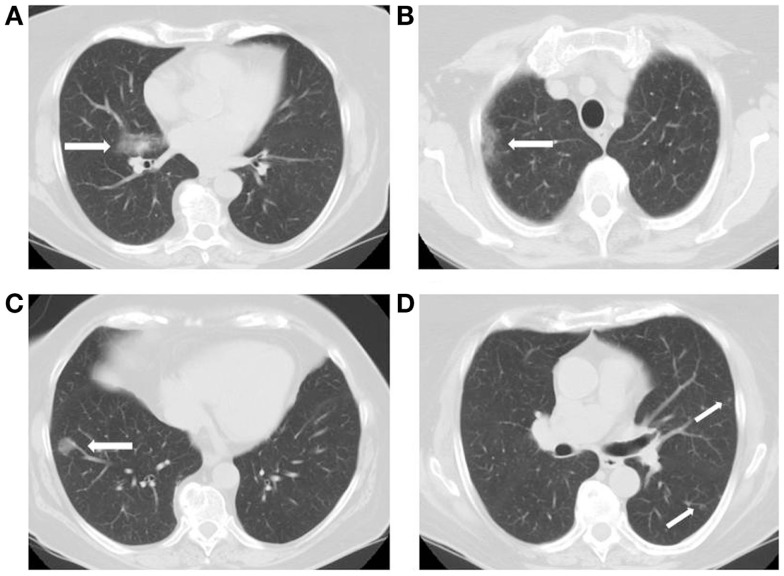
**DL (A) was resected concomitantly with lesions in (B,C) (final pathology = IA) while nodules in (D) were radiologically observed**.

A representative case is included in Figures 1A–D. This was a 71-year-old, female, patient presented in 2010 with RML DL (Figure 1A) as well additional bilateral pulmonary nodules (Figures 1B–D). DL was resected by open lobectomy while RUL (Figure 1B) and RLL (Figure 1C) nodules were wedged out during the same operation. At definitive pathology, these were all found to be invasive adenocarcinoma. Additional small nodules in the left lung (Figure 1D) were radiologically observed and they were stable at 43 months of follow-up.

After excluding nodules in the same lobe as the DL, the remaining nodules in G2 and all nodules in G3 were assessed by using a simplified Lung-RADS assessment and a modified version of the NCCN Guidelines (Table 6). In Group 2, the Lung-RADS system, placed 15/21 (71%) of the nodules in Category 2, which predicts a <1% probability of malignancy yet 4/15 nodules in this category were malignant. Lung-RADS correctly predicted the malignancy rate in Category 3, Category 4A, and Category 4B. Comparatively, “NCCN suggested malignancies” were found in 0/9 nodules in Category A, 3/4 in Category B, and 7/8 in Category C.

**Table 6:** **Nodules risk assessment by Lung-RADS and NCCN Guidelines**.

Model	Category	Group 2	Group 3	Enlarged lesions		
				Time 1	Time 2	Time 3
No. of nodules		21^a^	157	9	9	7^b^
Lung-RADS	2	15 (71%)	137 (87%)	7 (78%)	0	3 (33%)
	3	3 (14%)	13 (8%)	2 (22%)	0	0
	4A	1 (5%)	4 (3%)	0	7 (78%)	3 (33%)
	4B	2 (10%)	3 (2%)	0	2 (22%)	1 (11%)
NCCN-Guidelines	A	9 (43%)	113 (72%)	3 (33%)	0	3 (33%)
	B	4 (19%)	26 (17%)	3 (33%)	1 (11%)	0
	C	8 (38%)	18 (11%)	3 (33%)	8 (89%)	4 (44%)

*Time 1 = at baseline CT scan, Time 2 = when nodule was found to be enlarged, Time 3 = when additional diagnostic study was performed due to suspicion of lung cancer*.

*^a^Only nodules located in a different ipsilateral lobe as the DL or contralaterally*.

*^b^The remaining two lesions were still under radiological surveillance when the risk assessment was performed*.

In Group 3, Lung-RADS assessed a Category 2 risk to 137/157 (87%) nodules and NCCN assessed a Category A risk to 113/157 (72%) based on the CT prior to surgical resection of the DL. During surveillance, all but nine nodules remained stable and the nodules that were stable were down graded to a low risk category in each system. Risk assessment was performed and reported on the nodules that enlarged at three specific intervals of time as define in the methods (Table 6). After assessment at Time 2, the additional studies included short interval CT scan (6), PET-scan (1), antibiotic therapy (1), and percutaneous CT-guided biopsy (1). Four of seven lesions remained in the high risk category and they were all found to be malignant. The remaining three nodules were stable and then classified into low risk category.

## Discussion

The primary finding in this study is that nodules identified on pre operative imaging and followed after resection of the early stage dominant adenocarcinoma have a low rate of enlargement and only 2.5% become malignant at 3.2 years of follow-up. However, resected nodules found in the ipsilateral lobes separate from the DL have a rate of malignancy 48%. Nodules in the ipsilateral thorax, size, morphology, location, PET avidity, and shorter VDT are more concerning for malignancy. In addition, risk assessment with Lung-RADS or NCCN nodule guidelines offer reassurance of the importance of select non-dominant nodules that may benefit from treatment. Taken together, these data would support a strategy of surveillance of existing nodules after resection of the dominant adenocarcinoma.

The rate of malignancy in these secondary nodules in our series is similar to a recent analysis by Stiles and colleagues (16) who determined the fate of these nodules in a more diverse group of patients that included both adenocarcinoma and squamous carcinoma. They also demonstrated that 39% of nodules resected concomitantly with the DL will be malignant, but nodules surveyed after resection have a 4.8% rate of malignancy. This suggests that a reasonable surgical approach in the ipsilateral chest is to resect the known nodules found in the other lobes and address the contralateral nodules with surveillance. Supporting this hypothesis is the fact that ipsilateral location of the additional nodules was found to be an independent predictor of malignancy, compared to contralateral location (16). Additionally, this strategy allows the surgeon to make a definitive diagnosis of lesions suspicion for lung cancer during the same chest operation. However, without more predictive power than surgeon judgment the deep-seated lesion in a lobe separate from the DL in the ipsilateral chest remains a surgical challenge.

These results also suggest that there is some judgment by the surgeon during pre-operative evaluation and during surgery that deems these nodules to be important enough to consider resection. The surgical decision making is easier if these are peripheral lesions amenable to additional wedge resections in a patient with excellent pulmonary function. The decisions are more difficult with a deeply seated lesion, multiple nodules or when operating on a patient with limited pulmonary reserves. For appropriate lesions that we are concerned about, we consider a pre surgery navigational bronchoscopy or even transthoracic needle biopsy to help plan surgery at the current time.

During surgical planning, our results suggest that assessment of the non-dominant nodule(s) using the NCCN Guidelines may provide additional information. When the lesion was graded a Category B or C risk on the pre-operative scan 10/12 (83%) lesions were found to be malignant. Whereas, the assessment of risk by Lung-RADS at a single point in time does not seem to provide additional discrimination because this system relies in changes over time to determine the likelihood of malignancy. However, the size of the lesion is the primary indicator in the NCCN Guidelines (>6 mm) and in a larger series of patients may be prone to “error” with small lesions.

The application of both of these models during follow-up of the 157 observed nodules seems to be appropriate since the scenario is akin to lung cancer screening for which these two tools were developed. In this situation, both size of the lesion and an increase in the size of the lesion are key factors, which ultimately determine risk. For most surgeons, these two factors already enter into the evaluation of an existing pulmonary nodule. However, growth rate and PET avidity of the nodules have also been shown to represent important predictors of malignancy (17, 18), while the presence of a solid component generally correlate with an invasive adenocarcinoma (19). The value of continued surveillance and risk assessment after resection is emphasized by our data. Even though a number of nodules were assessed in the higher risk category, following the I-ELCAP algorithm for nodule management allowed many nodules to be down graded in concern while several persisted in importance.

Whether and how these two risk assessment tools can be integrated into our practice remains an ongoing process of evaluation. Surgeons have two needs: determining the risk of nodules while planning a surgical intervention and surveillance of nodules. One interpretation of our data may be that neither of these risk assessment tools was better than surgeon judgment in surgical planning but there remains a need to be able to more reliably predict malignancy since two studies show that half of nodules resected were benign. We believe that surveillance of nodules whether at screening or in cancer surveillance is a process for which our team has followed nodules using the I-ELCAP protocol for over a decade and this experience cannot be underestimated. However, an additional simplified tool for all team members may be useful and the ultimate benefit of these models during surveillance is that they provide a standardized tool for the whole thoracic oncology team (radiology, oncology, surgeon) to use rather than relying on one physician’s individual assessment for clinical assessment of the radiological findings. A prospective study to test the applicability to a non-screened cohort may be of use.

There are several limitations to our study. First, we retrospectively applied the risk assessment models to not only a predominant non-screened population, but also where we knew the final histology of the nodules. Further prospective study and analysis is required to ascertain if this is valid. Second, there was no mandated CT slice thickness protocol. Most of the patients had a standard dose chest CT scan with or without contrast but the slice thickness varied from 2.5 to 5 mm, which could alter the nodule counts and assessment. However, this is how patients present in clinical practice. Third, the duration of follow-up is relatively short at a median of 3.2 years. Even though we may consider solid nodules that are stable past 2 years of surveillance indolent, according to the Fleischner society (20), 42% of the nodules in our series undergoing surveillance were non-solid or part-solid and require a different management algorithm and a longer duration of follow-up (21). Despite the clinical indolent nature showed by nodules in Group 3, malignant histology should not be excluded since pathology is unknown for most of them. However, 72% of these nodules measured <6 mm in mean diameter and major lung cancer screening protocols would address to them a less intensive follow-up, by annual or 6-month CT scan (8, 9, 12), being the estimated risk of malignancy <1% (8).

In summary, surveillance of the remaining nodules after resection of the DL in patients with adenocarcinoma and a lepidic component associated with additional nodules is a reasonable strategy. These nodules exhibited a growth rate of only 6% and malignancy rate 2.5% in the 157 lesions for which follow-up strategy was undertaken. In contrast, nodules resected at the time of the DL harbor malignancy in 48% of the 21 nodules that were located in a different lobe from the DL. Application of Lung-RADS and NCCN risk categories may provide additional information about the importance of theses nodules particularly as a more standardized tool for the clinical assessment of multiple lung nodules but requires further study.

## Author Contributions

The authors declare: substantial contributions to the conception or design of the work; or the acquisition, analysis, or interpretation of data for the work. Drafting the work or revising it critically for important intellectual content. Final approval of the version to be published. Agreement to be accountable for all aspects of the work in ensuring that questions related to the accuracy or integrity of any part of the work are appropriately investigated and resolved.

## Conflict of Interest Statement

The authors declare that the research was conducted in the absence of any commercial or financial relationship that could be construed as a potential conflict of interest.
